# Downregulation of circ_0000673 Promotes Cell Proliferation and Migration in Endometriosis via the Mir-616-3p/PTEN Axis

**DOI:** 10.7150/ijms.63564

**Published:** 2021-08-17

**Authors:** Yongwen Yang, Deying Ban, Chun Zhang, Licong Shen

**Affiliations:** 1 Department of Clinical Laboratory, Xiangya Hospital, Central South University, No. 87 Xiangya Road, Changsha, 410008, P. R. China.; 2 Department of Gynecology, Xiangya Hospital, Central South University, No. 87 Xiangya Road, Changsha, 410008, P. R. China.

**Keywords:** circRNA, endometriosis, miRNA, PTEN

## Abstract

Endometriosis is a common gynecological disease, affecting up to 10% of women of reproductive age and approximately 50% of women with infertility. Circular RNAs (circRNAs) have been shown to be involved in a number of diseases. Dysregulated expression of circRNAs in endometriosis has been reported, and circ_0000673 was significantly downregulated. However, the details of its role in the pathogenesis of endometriosis are still poorly understood. We investigated the location and effects of the downregulation of circ_0000673 in endometriosis. We demonstrated that knockdown of circ_0000673 significantly increased the proliferation and migration of eutopic and normal endometrial cells. Bioinformatics analysis predicted that circ_0000673 might act as a sponge for miR-616-3p. We found that the effect of circ_0000673 knockdown could be recovered by miR-616-3p inhibitor and enhanced by miR-616-3p mimics. qPCR and western blot assays showed that circ_0000673 knockdown could decrease the expression of PTEN and increase the expression of PI3K and p-AKT. PTEN was confirmed to be a target of miR-616-3p. These results demonstrated that the downregulation of circ_0000673 could promote the progression of endometriosis by inactivating PTEN via the deregulation of miR-616-3p.

## Introduction

Endometriosis is a debilitating disorder that is caused by the presence of endometrial cells outside the uterine cavity. It affects up to 10% of women of reproductive age, affecting over 176 million women worldwide 1, 2. Approximately 50%-80% of women with endometriosis suffer from pelvic pain, and up to 50% suffer from infertility 1, 2. The exact etiology and pathophysiology of endometriosis is unclear. The most widely accepted hypothesis is that endometriosis is initiated by the transport of endometrial fragments into the pelvic cavity by retrograde menstruation. However, the development of ectopic lesions also depends on factors that facilitate the viability, proliferation, adhesion, neoangiogenesis, and migration of the reverse endometrial pieces and cells 2. However, the underlying molecular mechanisms need to be fully understood, to enable the exploration of new targets for the treatment of endometriosis.

Circular RNAs (circRNAs) are a class of endogenous RNAs with a closed loop structure that makes them more stable than linear RNAs. circRNAs are highly abundant and specifically expressed in different tissues and developmental stages and have been found to participate in the regulation of biological and pathological processes such as cell proliferation, apoptosis, invasion, angiogenesis, and the epithelial-mesenchymal transition (EMT), by acting as microRNA (miRNA) sponges 3-5. Previous studies have found aberrant circRNA expression in endometriosis 6, 7. Some of these studies showed that circRNAs promote the pathogenesis of endometriosis via regulating the function of miRNAs 8-10. In a previous work, we found that circ_0000673 in the ectopic endometrium (EC) was significantly downregulated compared to the eutopic endometrium (EU) 11. Circ_0000673 is encoded by the *RSL1D1* gene and is 251 bp long. The *RSL1D1* gene contributes to the cell cycle, cell proliferation, apoptosis, and metastasis, by downregulating the expression of the *PTEN* gene 12, 13. The dysregulation of circ_0000673 in neoplasms may act as a novel oncogene by regulating cell proliferation, migration, and invasion 14, 15.

Using bioinformatics analysis, we found that circ-0000673 contains an miR-616-3p response element. miR-616-3p is reported to be a carcinogenic miRNA (onco-miRNA) in ovarian cancer, breast cancer, and gastric cancer, acting by promoting cell proliferation, reducing apoptosis, and promoting cell invasion and metastasis, the EMT, and other processes. Dysregulated miR-616-3p contributes to abnormal proliferation and apoptosis by targeting PTEN in hepatocellular carcinoma, renal tubular epithelial cells, and cardiomyocytes 16, 17. However, the functions of circ_0000673 and miR-616-3p in endometriosis have not been clarified. The aim of the present study was to explore the potential role of circ_0000673 in the pathogenesis of endometriosis.

## Materials and methods

### Clinical samples

This study was approved and supervised by the Medical Ethics Committee of Xiangya Hospital, Central South University. Written informed consent was obtained from all subjects. Twenty patients with ovarian endometriomas at stage III-IV who provided both EU and EC and 20 women without endometriosis, as normal endometrial (NE) controls, were recruited during the proliferative phase. All subjects were 20-45 years old, with regular menstrual cycles, and had received steroid hormone treatment at least 3 months before specimen collection.

### Fluorescent *in situ* hybridization

The EU and EC tissues were embedded in paraffin and sliced. Slices were dewaxed in xylene, dehydrated in anhydrous ethanol, and then treated with protease K (20 µg/mL). Subsequently, 3% methanol-H_2_O_2_ was added to block endogenous peroxidase. After prehybridization, the hybridization solution with a digoxigenin (DIG) -labeled probe was added, and the sections were hybridized overnight at 42 °C. The sections were then incubated with mouse anti-digoxigenin-labeled peroxidase (anti-DIG-HRP; Jackson ImmunoResearch, West Grove, PA, USA) at 37 °C for 50 min. After washing three times, freshly prepared FITC-TSA chromogenic reagent (Servicebio, Wuhan, China) was added to the reaction in the dark for 5 min at room temperature, and then the sections were incubated with DAPI for 5 min. Finally, the slices were sealed and observed using a Nikon Laser Scanning Confocal Microscope (Nikon, Tokyo, Japan).

### Cell culture and identification

The endometrial tissue was rinsed with Hanks' solution and cut into pieces in F12/DMEM culture medium, followed by collagenase digestion for 60 min and DNase I digestion for 30 min. After centrifugation, the cells were suspended in F12/DMEM with 10% fetal bovine serum and filtered through a 70-μm cell strainer to collect endometrial stromal cells. The endometrial stromal cells were inoculated in F12/DMEM with 10% fetal bovine serum in an incubator with 5% CO_2_ at 37 °C. The isolated endometrial stromal cells were identified by staining with vimentin using immunofluorescent techniques as previously described 18.

### Cell transfection

Primary eutopic and normal endometrial stromal cells were treated at the third passage, with a cell density of 1 × 10^4^ mL. Lentivirus vectors (GenePharma, Shanghai, China) targeting the junction region of hsa_circ_0000673 were constructed to knockdown the expression of hsa_circ_0000673 in endometrial stromal cells. All lentivirus vectors were transfected into eutopic and normal endometrial stromal cells with transfection enhancers. The sequences were as follows: LV-1, 5′-GTGGTTCTTGCAGATTATCTC-3′; LV-2, 5′-CTTGCAGATTATCTCCCTCCA-3′; LV-negative control (NC), 5′-TTCTCCGAACGTGTCACGT-3′. The transfection efficiency was confirmed using fluorescence microscopy after 24 h, and cells were harvested for further functional experiments after 48 h. Has-miR-616-3p mimic or inhibitor (RiboBio, Guangzhou, China) was transfected using Lipofectamine 2000 reagents (Invitrogen, Carlsbad, CA, USA) according to the manufacturer's protocol. All experiments were performed three times in each assay.

### CCK-8 assay

The CCK-8 assay was carried out using Cell Counting Kit-8 (NCM Biotech, Suzhou, China) according to the manufacturer's protocol. Briefly, cells were seeded into 96-well plates and divided into four groups, with four duplicate wells in each group. After cell attachment, the lentivirus vectors were added, and the plates were incubated at 37 °C for 24 h. 10 μL CCK-8 reagent was added into each well, and the plates were incubated at 37 °C for 2 h. Cell proliferation was determined by testing the absorbance value at 450 nm using a spectrophotometer.

### Colony formation assay

Cells were placed in six-well plates with 1000 cells per well. LV-circ_0000673 or miR-616-3p mimic/inhibitor was transfected, and the cells were cultured for 2 weeks. After washing, the cells were successively treated with 4% paraformaldehyde and 0.1% crystal violet. The number of colonies in each well was counted under the microscope.

### Wound healing assay

Wound healing assays were performed to assess the migration capability of endometrial stroma cells after knockdown of circ_000673. Briefly, endometrial stromal cells were seeded in six-well plates, and LV-circ_0000673 or miR-616-3p mimic/inhibitor was transfected. Then, the monolayered cells were scraped with a micropipette tip to make a straight wound and cultured with serum-free medium for 24 h. The gap width at 0 and 24 h was recorded using microscopy.

### qRT-PCR

Cells were collected 72 h after transfection, and total RNA was extracted using TRIzol reagent. The concentration and purity of the RNA were measured using a NanoVue Plus spectrophotometer (Healthcare Bio-Science AB, Uppsala, Sweden). The primers were listed in Table 1. miR-616-3p stem loop reverse transcription primers and U6 control were designed and synthesized by Guangzhou Ruibo Biological Co., Ltd. Reverse transcriptase kits (Takara Bio Inc., Shiga, Japan) were used for the reverse transcriptional reaction. Gene expression was performed with SYBR Green qPCR mix (Bio-Rad, Hercules, CA, USA) using Applied Biosystems 7900 Real-Time PCR system (Applied Biosystems, Foster City, CA). Relative gene expression was analyzed using the 2^-ΔΔCt^ method.

### Western blot assays

Endometrial cells were lysed in RIPA lysis buffer to extract total protein. Aliquots of 50 μg total protein were taken for 10% SDS-PAGE electrophoresis, transferred onto a 0.45-μm PVDF membrane, and blocked with 5% nonfat milk. Primary antibodies of PTEN (1:1000, Abcam, Cambridge, UK), PI3K (1:1000, Abcam, Cambridge, UK), p-AKT (1:2000, CST, Danvers, USA), and GAPDH (1:1000, Abcam, Cambridge, UK) were added and incubated overnight at 4 °C. Then, after washing for three times, the secondary antibodies were added and incubated at room temperature for 1 h. Finally, enhanced chemiluminescence reagent (CST, USA) was added, and the reactivity was determined by an enhanced chemiluminescence detection system (Amersham Biosciences, Pittsburgh, PA, USA).

### Dual luciferase assays

Dual luciferase assays were performed to assess the binding between circ_0000673, PTEN, and miR-616-3p. The wild-type (WT) circ_0000673 and the PTEN 3′-UTR were inserted into pMIR-reporter plasmids (OBio Technology Corp, Shanghai, China), and the mutant (MUT) type was designed by mutating the binding site of the seed sequence. The WT and MUT luciferase reporter plasmids were co-transfected with miR-616-3p mimics or negative control into 293T cells. The relative luciferase activity was measured using luciferase reporter kits (Promega, Madison, WI, USA).

### Statistical analysis

Data were expressed as mean ± standard deviation (SD). Two-tailed Student's *t* tests were performed for pairwise comparisons, and the one-way analysis of variance was used for multiple comparisons. All experiments were performed in triplicate, and a P value <0.05 was taken to indicate statistical significance. The statistical analysis was conducted using SPSS 25.0 and GraphPad Prism 9.0 (GraphPad Software, San Diego, California USA, www.graphpad.com).

## Results

### Decreased expression of circ_0000673 in endometriosis

**Figure 1:**
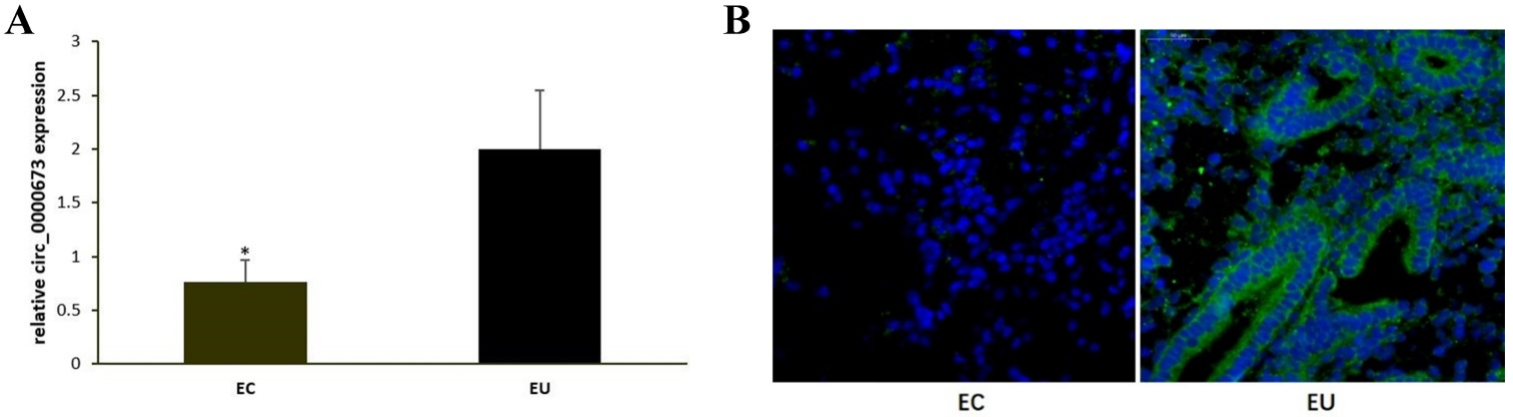
** Hsa_circ_0000673 was downregulated in ectopic endometrium compared to eutopic endometrium. (A)** hsa_circ_0000673 expression in paired EC and EU was detected by qPCR analysis. **(B)** hsa_circ_0000673 expression was examined by fluorescent *in situ* hybridization. Blue is the nucleus, and green represents the positive expression of hsa_circ_0000673. * *P*< 0.05.

qPCR confirmed that circ_0000673 was significantly downregulated in EC compared with EU (Figure 1A). Fluorescent *in situ* hybridization also showed that circ_0000673 was expressed at low levels or not expressed in EC, whereas it was upregulated in EU. It was mainly distributed in the cytoplasm (Figure 1B).

### Knockdown of circ_0000673 promotes endometrial cell proliferation and migration

**Figure 2:**
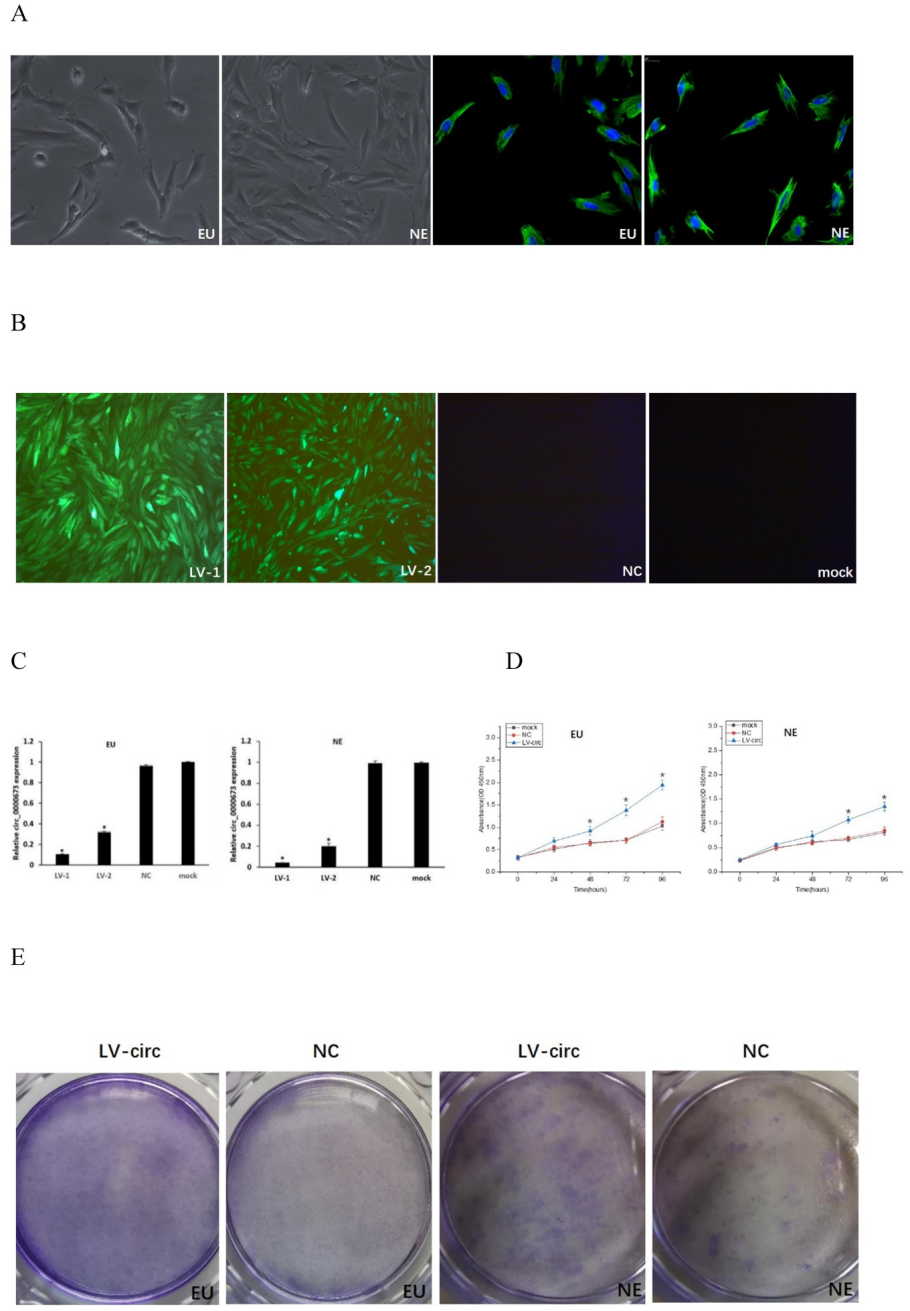

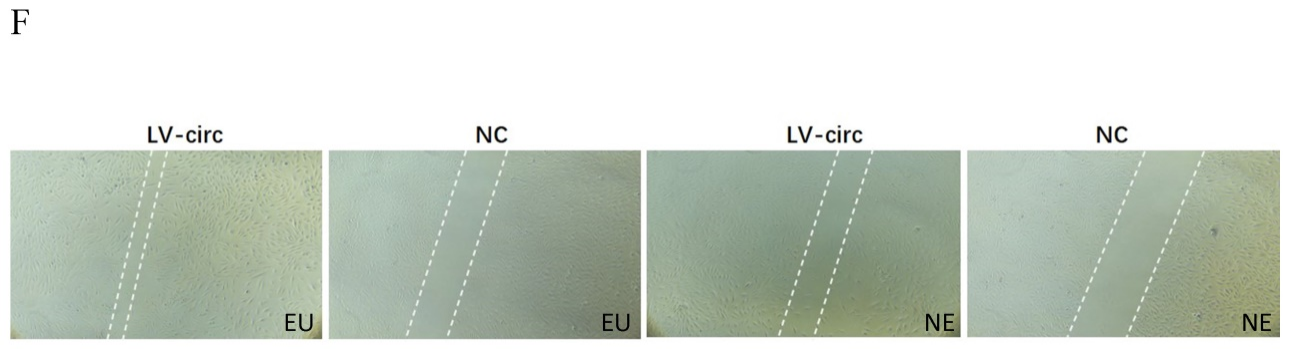
** Knockdown of circ_0000673 promoted endometrial cell proliferation and migration. (**A) Identification of endometrial stromal cells. Endometrial stromal cells presented a fusiform cell morphology. Vimentin-positive expression by immunofluorescence staining confirmed the presence of endometrial stromal cells. **(B)** The fluorescence of green fluorescent protein was used to evaluate transfection efficiency and showed that the cells were successfully transfected by circ_0000673 lentiviral vectors (green). Blue represents the nuclei. **(C)** qPCR confirmed significant downregulation of circ_0000673 after transfection with LV-circ_0000673-1/2 in endometrial stroma cells. **(D)** The CCK-8 experiment showed that the cell proliferation abilities of endometrial stromal cells were significantly increased after circ_0000673 knockdown. **(E)** Clone formation assays demonstrated that cell vitality in the group transfected with circ_0000673 lentiviral vectors was higher than that in the control group. **(F)** Wound healing assays showed that the cell migration capacity was enhanced by circ_0000673 knockdown. * Mean compared with negative control group, *P* < 0.05.

Endometrial stromal cells presented a fusiform cell morphology under light microscopy, and the positive expression of vimentin by immunofluorescence stain confirmed the presence of endometrial stromal cells (Figure 2A). Transfection efficiency was observed from the fluorescence of green fluorescent protein, which demonstrated that the cells were successfully transfected with circ_0000673 knockdown lentiviral vectors (Figure 2B). qPCR confirmed that circ_0000673 was expressed at significantly lower levels after transfection with LV-circ_0000673-1/2 in endometrial stromal cells (Figure 2C). CCK-8 and cell clone formation assays were performed to assess the function of circ_0000673 in endometrial cell proliferation. The cell proliferation rate and colony formation capacity in the knockdown LV-circ group were significantly increased compared with those of the negative control (Figure 2D, E). These results indicated that the downregulation of circ_0000673 may play important roles in cell proliferation in endometriosis. Wound healing assays showed that the numbers of migrating cells after transfection with circ_0000673 knockdown lentiviral vectors were significantly increased compared with those in the control group (Figure 2F).

### circ_0000673 targets miR-616-3p

**Figure 3:**
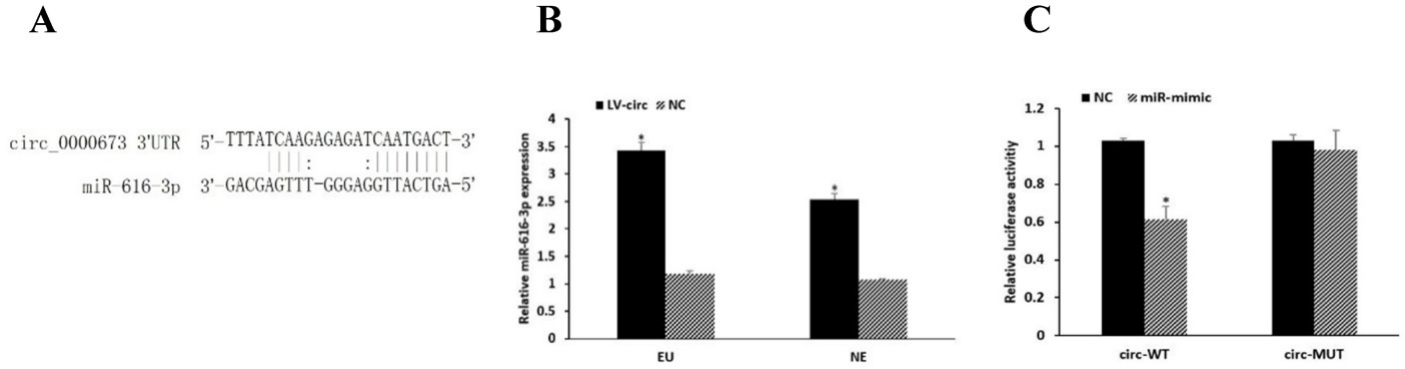
**Circ_0000673 suppressed the expression of miR-616-3p. (A)** The binding site of circ_0000673 and miR-616-3p. **(B)** qPCR showed that the miR-616-3p expression was significantly increased after circ_0000673 knockdown in endometrial cells. **(C)** Relative luciferase activity was attenuated in the group transfected with circ-WT and miR-mimic, whereas no differences were observed in those co-transfected with mutant plasmids or negative control. Data are shown as mean ± SD. *Mean compared with negative control group, *P* < 0.05.

circRNAs have been known to be involved in regulating gene expression by serving as miRNA sponges. We predicted circRNA-miRNA interactions using the TargetScan 19 and miRanda 20 databases, which revealed that miR-616-3p has a binding site to circ_0000673 (Figure 3A). The expression of miR-616-3p was significantly increased after circ_0000673 knockdown (Figure 3B). Compared with the negative group, luciferase intensity was significantly reduced in the group co-transfected with circ_0000673-WT plasmids and miR-616-3p mimics, whereas no significant differences in luciferase intensity were observed in those co-transfected with mutant plasmids or negative controls (Figure 3C). The data verified the binding between circ_0000673 and miR-616-3p.

### circ_0000673/miR-616-3p induces endometrial cell proliferation and migration by repressing PTEN

**Figure 4:**
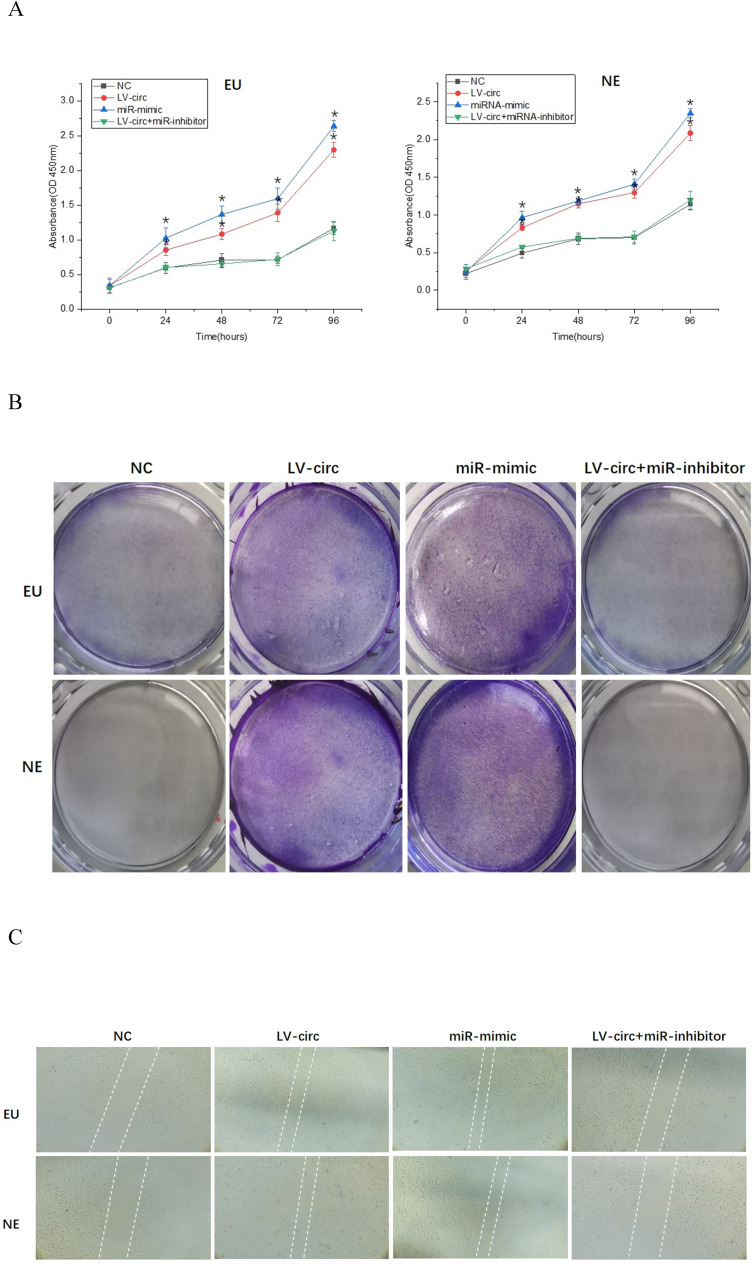

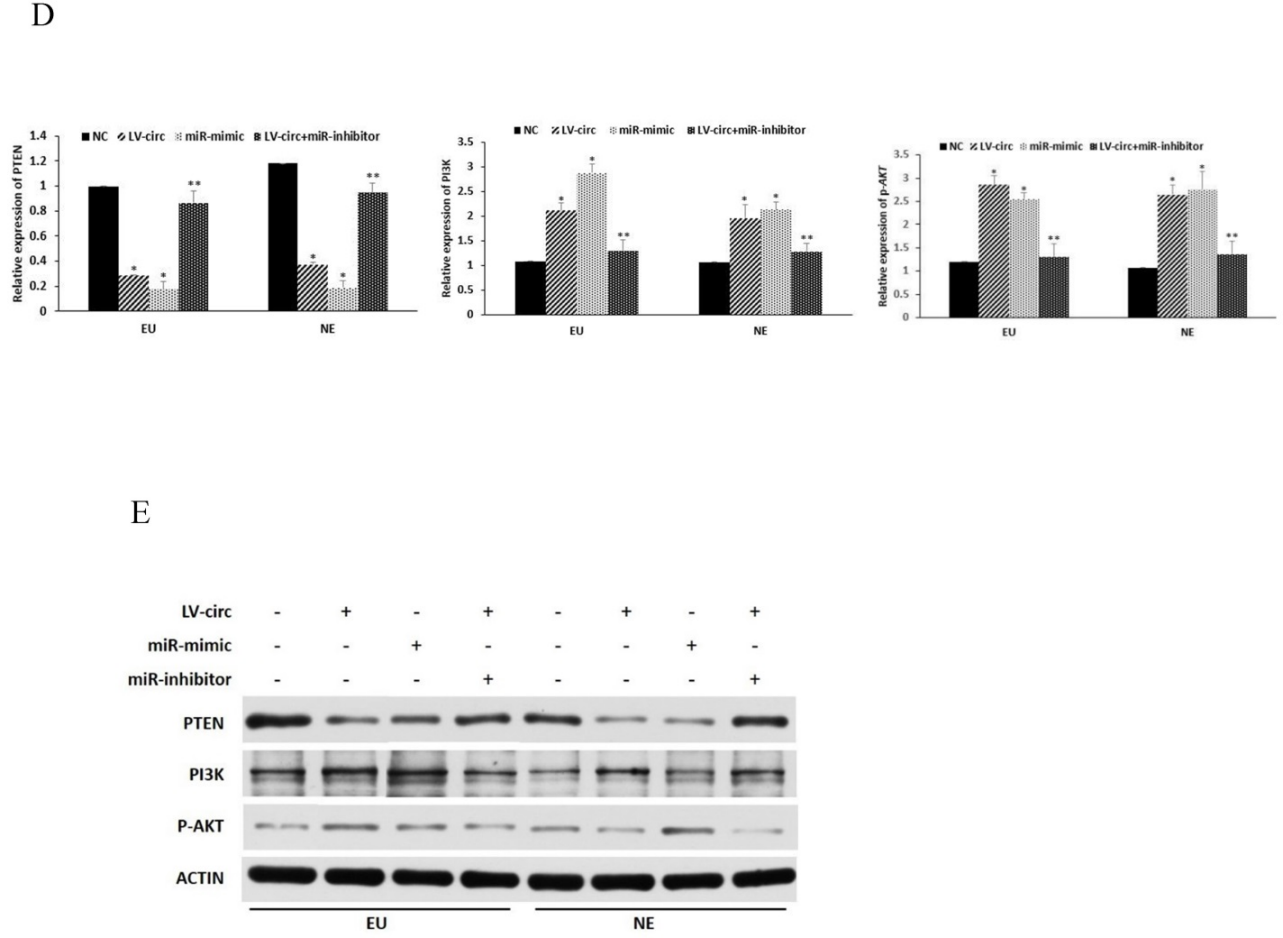
** Effect of circ_0000673/miR-616-3p on cell proliferation and migration via PTEN. (A)** CCK-8 experiments were performed to assess the proliferation capacity of EU/NE cells treated with negative control, LV-circ, miR-mimic, or LV-circ+miR-inhibitor. **(B)** Colony formation assays were performed to detect the vitality of EU/NE cells treated with negative control, LV-circ, miR-mimic, or LV-circ+miR-inhibitor. **(C)** Wound healing assays were conducted to identify the migration capacity of EU/NE cells treated with negative control, LV-circ, miR-mimic, or LV-circ+miR-inhibitor. **(D)** qPCR was performed to examine the relative mRNA levels of *PTEN*, *PI3K*, and p-*AKT* in EU/NE cells treated with negative control, LV-circ, miR-mimic, or LV-circ+miR-inhibitor. **(E)** Western blot assay was used to detect the protein expression of PTEN, PI3K, and p-AKT in EU/NE cells treated with negative control, LV-circ, miR-mimic, or LV-circ+miR-inhibitor. *Means compared with negative control group, *P* < 0.05. **Means compared with LV-circ group, *P* < 0.05.

To further clarify the mechanisms of circ_0000673/miR-616-3p with respect to endometrial cell proliferation and migration, cells were transfected with circ_0000673 lentivirus knockdown vectors and/or miR-616-3p mimics/inhibitors. Cells transfected with knockdown LV-circ_00007673 or miR-616-3p mimic showed significantly increased proliferation capacity, according to CCK-8 and colony formation assays (Figure 4A, B), and also increased migration capacity according to wound healing assays (Figure 4C). The increased capacity for proliferation and migration was recovered by adding an miR-616-3p inhibitor (Figure 4A, B, C). qPCR and western blot assays revealed that circ_00007673 knockdown or miR-616-3p mimics reduced the expression of PTEN and increased PI3K and p-AKT. The expression of PTEN, PI3K, and p-AKT was recovered by adding miR-616-3p inhibitor (Figure 4D, E). These data showed that circ_00007673 and miR-616-3p played opposite roles in endometriosis and downregulation of circ_0000673 increased the expression of miR-616-3p, which may facilitate the downregulation of PTEN by miR-616-3p.

### PTEN is a target of miR-616-3p

**Figure 5:**
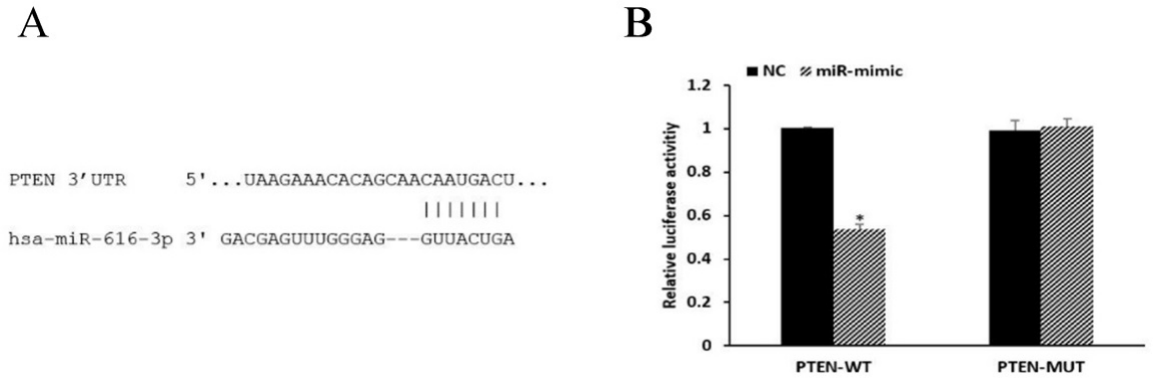
**PTEN was a target of miR-616-3p. (A)** The binding site of miR-616-3p and PTEN. **(B)** Relative luciferase activity was statistically significantly reduced in the group transfected with PTEN-WT and miR-mimic, and no changes were observed in those treated with PTEN-MUT or negative control. Data are shown as mean ± SD. *Mean compared with negative control group, *P* < 0.05.

miRNAs are known to be important post-transcriptional repressors, acting by binding to the 3′-UTR. We conducted luciferase reporter assays to assess whether the expression of PTEN was directly regulated by miR-616-3p. miR-616-3p mimics significantly reduced the luciferase activity when co-transfected with PTEN-WT plasmids, whereas no effect on luciferase activity was observed when they were co-transfected with PTEN-MUT plasmids. These data confirmed that miR-616-3p targeted PTEN (Figure 5).

## Discussion

Endometriosis is an enigmatic disease, affecting mainly women of reproductive age and causing pain and reduced fertility. The treatments available are sub-optimal because of the uncertainty of the pathogenesis of endometriosis 21, 22. Exploring these mechanisms and identifying new molecular targets are needed as a basis for the development of more effective treatments for endometriosis. circRNAs have been reported to be important in regulating gene expression and biological processes. Differential expression of circRNAs in endometriosis has been identified 6, 11, but the underlying mechanisms have not been clearly defined. In this study, we explored the function of a downregulated circRNA, circ_0000673, on proliferation and migration of endometrial tissue in endometriosis.

The proliferation and migration of endometrial tissue outside of the uterus is a defining characteristic of endometriosis, although the details of the pathological progression remain largely unknown 23. CircRNAs have been recognized as important molecules in various biological processes, and abnormalities in their abundance or function may contribute to many diseases 3, 24, 25. Studies have shown the abnormal expression of circRNAs in endometriosis and indicated their value in the diagnosis of diseases 26, 27. Our previous study showed the profile of differentially expressed circRNAs and revealed that circ_0000673 was attenuated in ovarian endometrioma 11. In this study, we confirmed the downregulation of hsa_circ_0000673 in the ovarian EC using fluorescent *in situ* hybridization, a finding that implied that it plays a role in ovarian endometriosis. Our findings indicate that loss of circ_0000673 could promote the proliferation and migration of endometrial cells. Recent studies have suggested that circATRNL1 promotes the EMT by regulating Yes-associated protein 1 28, and that high expression of circ_0007331 plays a vital role in the proliferation and invasion of cells in endometriosis 10. These studies suggest that circRNAs play a regulatory role in the progression of endometriosis. Recently, the dysregulation of circ_0000673 was reported to be involved in tumor invasion and differentiation in cholangiocarcinoma, indicating that it may act as a novel oncogene 14. We therefore hypothesized that circ_0000673 could be involved in regulating endometriosis.

circRNAs have been known to play important roles in miRNA targeting. We found that circ_0000673 could bind to miR-616-3p, which may target PTEN. The levels of PTEN are reduced in endometriosis and associated with the stage of the condition 29. Wu et al. revealed that miR-616-3p could facilitate the migration and EMT of gastric cancer cell lines 16. Thus, we hypothesized that downregulated circ_0000673 may promote the progression of endometriosis by upregulating miR-616-3p. We found that miR-616-3p was increased after circ_000073 knockdown. Dual luciferase assays identified miR-616-3p as a target of circ_0000673. The miR-616-3p mimic has a similar promoting effect on the proliferation and migration of endometrial cells as downregulation of circ_0000673, whereas the miR-616-3p inhibitor reversed the promoting phenomenon. These data confirmed that circ_0000673 was involved in regulating the proliferation and migration of endometrial cells via miR-616-3p.

The role of miRNAs is the degradation or inhibition of gene translation by binding to the 3′-UTR of the target gene. We found that PTEN was predicted to be the target of miR-616-3p and the direct binding relationship was confirmed by dual luciferase assays. PTEN is a tumor-suppressor gene with specific phosphatase activity and plays important roles in cell growth, proliferation, and migration via the PI3K-AKT pathway 30, 31. Studies have shown that PTEN expression was decreased both in endometriosis tissue and in primary cultured endometrial stomal cells, which contributed to cell proliferation 32. Aberrant PTEN expression in endometriotic stromal cells reduced cell apoptosis via AKT/mTOR signaling 33. In our study, the knockdown of circ_0000673 or the addition of the mimic of miR-616-3p reduced the mRNA and protein levels of PTEN in both eutopic and normal endometrial stromal cells, accompanied with the promotion of cell proliferation and migration. Inhibition of miR-616-3p in endometrial stromal cells alleviated the phenomenon of cell proliferation and migration. The activation of PI3K and AKT by abnormal PTEN expression plays important roles in the cell cycle and the establishment of endometriosis 34, 35. Our results showed increased expression of PI3K and p-AKT, with reduced expression of PTEN, in endometrial stromal cells with circ_0000673 knockdown. These results indicate that circ_0000673 is involved in the progression of endometriosis by promoting cell proliferation and the migration of endometrial stromal cells through mediation of the PTEN/PI3K/AKT signaling pathway and miR-616-3p.

## Conclusions

The dysregulation of circRNAs has been implicated in the pathology of several diseases, and decreases in the abundance of the circRNA circ_0000673 have been observed in endometriosis. Our study investigated the location and downregulation of circ_0000673 in tissues from patients with endometriosis, and the effects of its downregulation. We found the downregulation of circ_0000673 increased the expression of miR-616-3p and further blocked the target, PTEN, which then activated PI3K and p-AKT and promoted the proliferation and migration of endometrial stromal cells. Meanwhile, inhibiting miR-616-3p increased the expression of PTEN, and attenuated the cell proliferation and migration. The study revealed the roles of circRNAs in endometriosis, which may bring new approaches to exploring novel therapeutic targets for endometriosis.

## Figures and Tables

**Table 1:** Primers for qRT-PCR

Primers	Sequence (5′- 3′)
hsa_circ_0000673-F	ATCTGTAAACCTTCTGTCCAAGA
hsa_circ_0000673-R	TCAAAACTGCTCAGAAGGCG
PTEN-F	ACTATTCCCAGTCAGAGGCG
PTEN-R	TCACCTTTAGCTGGCAGACC
PI3K-F	GCACCTGAATAGGCAAGTC
PI3K-R	TCGCACCACCTCAATAAGT
AKT-F	GTGGAGGACCAGATGATGC
AKT-R	TGCCCCTGCTATGTGTAAG
GAPDH-F	TGCACCACCAACTGCTTAGC
GAPDH-R	GGCATGGACTGTGGTCATGAG
